# Edge roughness quantifies impact of physician variation on training and performance of deep learning auto-segmentation models for the esophagus

**DOI:** 10.1038/s41598-023-50382-z

**Published:** 2024-01-30

**Authors:** Yujie Yan, Christopher Kehayias, John He, Hugo J. W. L. Aerts, Kelly J. Fitzgerald, Benjamin H. Kann, David E. Kozono, Christian V. Guthier, Raymond H. Mak

**Affiliations:** 1 Department of Radiation Oncology, Brigham and Women’s Hospital, Dana-Farber Cancer Institute, Harvard Medical School, Boston, MA USA; 2 Artificial Intelligence in Medicine (AIM) Program, Mass General Brigham, Harvard Medical School, Boston, MA USA

**Keywords:** Machine learning, Three-dimensional imaging, Translational research

## Abstract

Manual segmentation of tumors and organs-at-risk (OAR) in 3D imaging for radiation-therapy planning is time-consuming and subject to variation between different observers. Artificial intelligence (AI) can assist with segmentation, but challenges exist in ensuring high-quality segmentation, especially for small, variable structures, such as the esophagus. We investigated the effect of variation in segmentation quality and style of physicians for training deep-learning models for esophagus segmentation and proposed a new metric, edge roughness, for evaluating/quantifying slice-to-slice inconsistency. This study includes a real-world cohort of 394 patients who each received radiation therapy (mainly for lung cancer). Segmentation of the esophagus was performed by 8 physicians as part of routine clinical care. We evaluated manual segmentation by comparing the length and edge roughness of segmentations among physicians to analyze inconsistencies. We trained eight multiple- and individual-physician segmentation models in total, based on U-Net architectures and residual backbones. We used the volumetric Dice coefficient to measure the performance for each model. We proposed a metric, edge roughness, to quantify the shift of segmentation among adjacent slices by calculating the curvature of edges of the 2D sagittal- and coronal-view projections. The auto-segmentation model trained on multiple physicians (MD1-7) achieved the highest mean Dice of 73.7 ± 14.8%. The individual-physician model (MD7) with the highest edge roughness (mean ± SD: 0.106 ± 0.016) demonstrated significantly lower volumetric Dice for test cases compared with other individual models (MD7: 58.5 ± 15.8%, MD6: 67.1 ± 16.8%, *p* < 0.001). A multiple-physician model trained after removing the MD7 data resulted in fewer outliers (e.g., Dice ≤ 40%: 4 cases for MD1-6, 7 cases for MD1-7, N_total_ = 394). While we initially detected this pattern in a single clinician, we validated the edge roughness metric across the entire dataset. The model trained with the lowest-quantile edge roughness (MD^ER^-Q1, N_train_ = 62) achieved significantly higher Dice (N_test_ = 270) than the model trained with the highest-quantile ones (MD^ER^-Q4, N_train_ = 62) (MD^ER^-Q1: 67.8 ± 14.8%, MD^ER^-Q4: 62.8 ± 15.7%, *p* < 0.001). This study demonstrates that there is significant variation in style and quality in manual segmentations in clinical care, and that training AI auto-segmentation algorithms from real-world, clinical datasets may result in unexpectedly under-performing algorithms with the inclusion of outliers. Importantly, this study provides a novel evaluation metric, edge roughness, to quantify physician variation in segmentation which will allow developers to filter clinical training data to optimize model performance.

## Introduction

Radiation therapy (RT), as a cancer treatment, is used in approximately 50% of cancer cases^1^. As part of constructing a RT plan, it is essential to segment tumors that need to be treated with high-dose radiation, and to also segment the organs and healthy tissues that need to be protected from radiation exposure. These healthy tissues are referred to as Organs at Risk (OAR). The current workflow of RT planning involves manual segmentation of tumors and OAR on 3-dimensional (3D) imaging such as computed tomography (CT) by trained professionals. This manual segmentation task is typically performed on an axial slice-by-slice basis, which sums into a 3D volumetric structure. This task can be time-consuming and is subject to high levels of variation^2–4^. More recently, Artificial Intelligence (AI) assisted segmentation of tumors and OAR in real-world datasets, especially deep-learning-based auto-segmentation, has emerged and shown its potential to streamline the RT planning process^3–7^. However, there are clear challenges in ensuring that AI deployment in the clinic will result in higher quality segmentations^5–8^. An important challenge is that variability in manually segmentations, including clinician errors, in real-world datasets may result in under-performance in AI auto-segmentation algorithms. The impact of highly variable, lower quality manual segmentations on training may be especially important with small organs with anatomic variability, such as the esophagus.

The esophagus is an essential OAR due to its radiosensitive mucosa but challenging to segment^9^. Without high contrast to neighboring mediastinal structures, the esophagus is difficult to segment on CT scans. Furthermore, prior studies have shown that trained experts using standardized segmentation protocols are still prone to inter- and intra-observer variability, due to their different experiences, personal preferences, and training backgrounds^10^.

Inconsistency in training segmentation can be one major factor that results in the suboptimal performance of AI segmentation models^7,8^. Substantial variation exists in segmentations generated in the clinic and those variation may be stylistic, or due to clinically meaningful variation in human performance^10–12^. To try to account for this variation, Balagopal et al.^10^ proposed a segmentation network for prostate cancer that can adapt to different physicians’ segmentations with various styles to improve performances. Hosny et al.^6^suggested interobserver variation may be due to physician preference and experience in a clinical validation study for auto-segmentation of non-small-cell lung cancer tumors using deep learning, which demonstrated lower satisfaction with segmentations produced by other clinicians than AI-generated segmentations.

However, few studies have explored the effect of clinical segmentation variability on algorithm training, and defined methods to quantify and identify meaningful variation at the individual clinician level. Most of the work to date regarding esophageal segmentation has focused on improving the overall performance of segmentation models by proposing innovative model architectures and evaluating methods using a limited number of test cases^13,14^. Their training data were acquired directly from the clinic without standardization in terms of segmentation inconsistency^13–16^.

**Figure 1:**
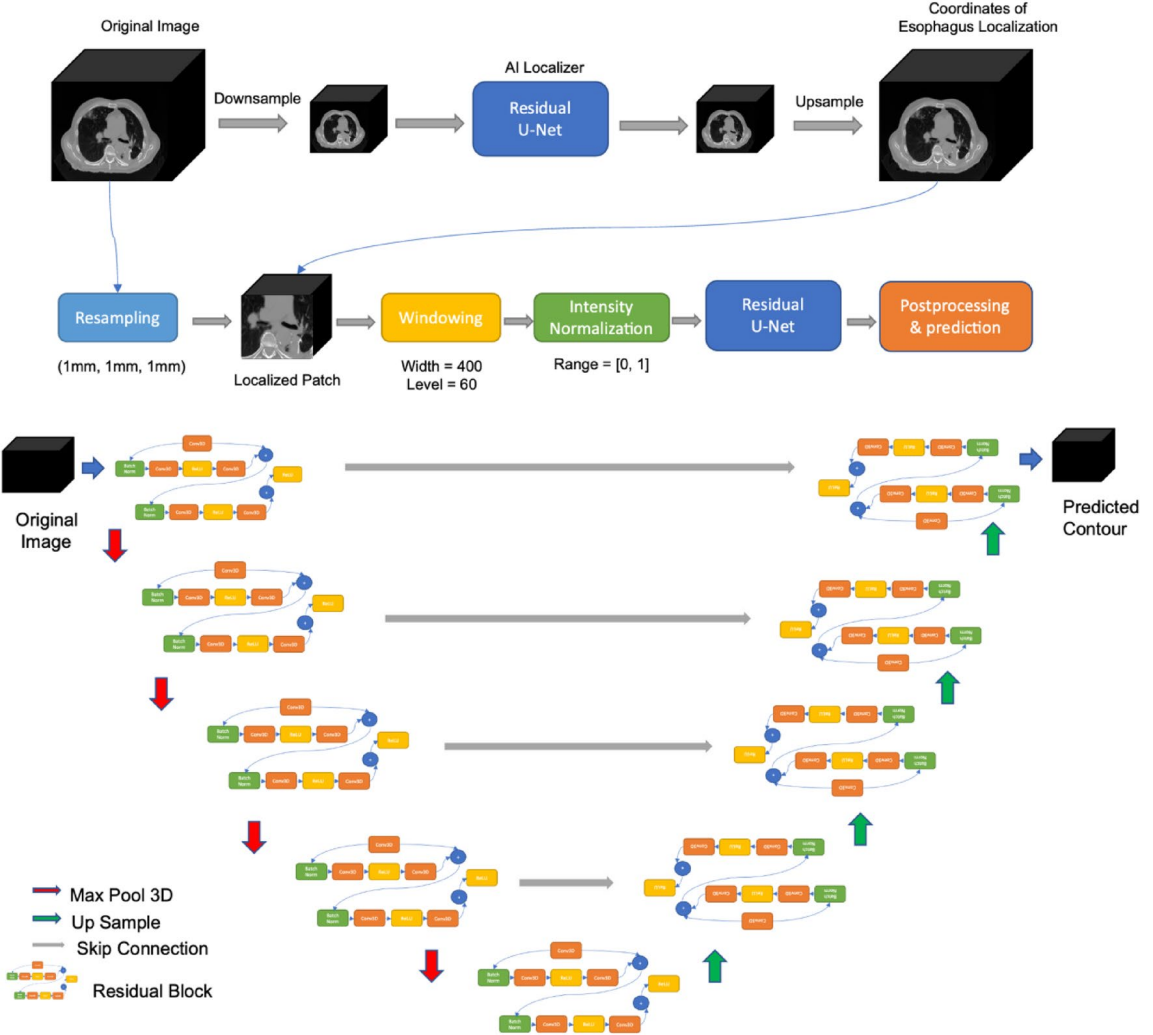
Methodology Workflow and Residual U-Net Architecture.

In this work, we investigated the effect of variation in segmentation quality and style of physicians on the development of deep-learning models for the 3D segmentation of the esophagus. We demonstrated variation in esophagus segmentation length between clinicians and more importantly, slice-to-slice inconsistency within a case by a given clinician for which we developed a new inconsistency metric defined by edge roughness, a curvature-based metric to quantify the shift of segmentation among adjacent axial slices of the CT image. We hypothesized that large edge roughness can introduce inaccuracy in segmentation model performance, and trained 3D U-Net models (Fig. 1) using both individual physician’s segmentations and combined segmentations from multiple physicians with differential edge roughness to test this hypothesis.

## Results

### Characteristics of study cohort

The cohort consisted of 394 patients (mainly lung-cancer patients) who received radiation therapy, each of which had a 3D CT image and esophagus segmentation. Contours were generated by 8 individual attending physicians. Demographic and treatment-related information of the cohort are summarized in Table 1. The median age was 71 and the most common cancer type was lung cancer. 55.3% of the patients were male and 44.7% were female. The median length of esophagus segmentations was 20 cm with a wide range from 3.6 cm to 27.0 cm. 86% of patients were treated during 2021 and 2022. More than 94% of the patients were treated with VMAT/IMRT.

**Table 1:** Characteristics of the Patient Cohort.

Characteristics	Number of Cases (n = 394)	
Age (year)	Median (25%, 75%)	71 (65, 80)
	Median (range)	71 (22, 95)
Gender	Male	218 (55.3%)
	Female	176 (44.7%)
Cancer type	Lung	387 (98.2)
	Other Secondary	5 (1.3%)
	Head and Neck	2 (0.5%)
Year of treatment	2022	193 (49.0%)
	2021	146 (37.0%)
	2020	54 (13.7%)
	2018	1 (0.3%)
Type of radiation treatment	VMAT/IMRT*	187 (47.5%)
	SBRT*	185 (46.9%)
	3D-Conformal	22 (5.6%)
Length of esophagus segmentation (centimeter)	Median (25%, 75%)	20.0 (11.0, 22.6)
	Median (range)	20.0 (3.6, 27.0)

*SBRT: Stereotactic body radiation therapy; VMAT: Volumetric modulated arc therapy; IMRT: Intensity-modulated radiation therapy.

### Descriptive statistics of length of esophagus segmentation

**Figure 2:**
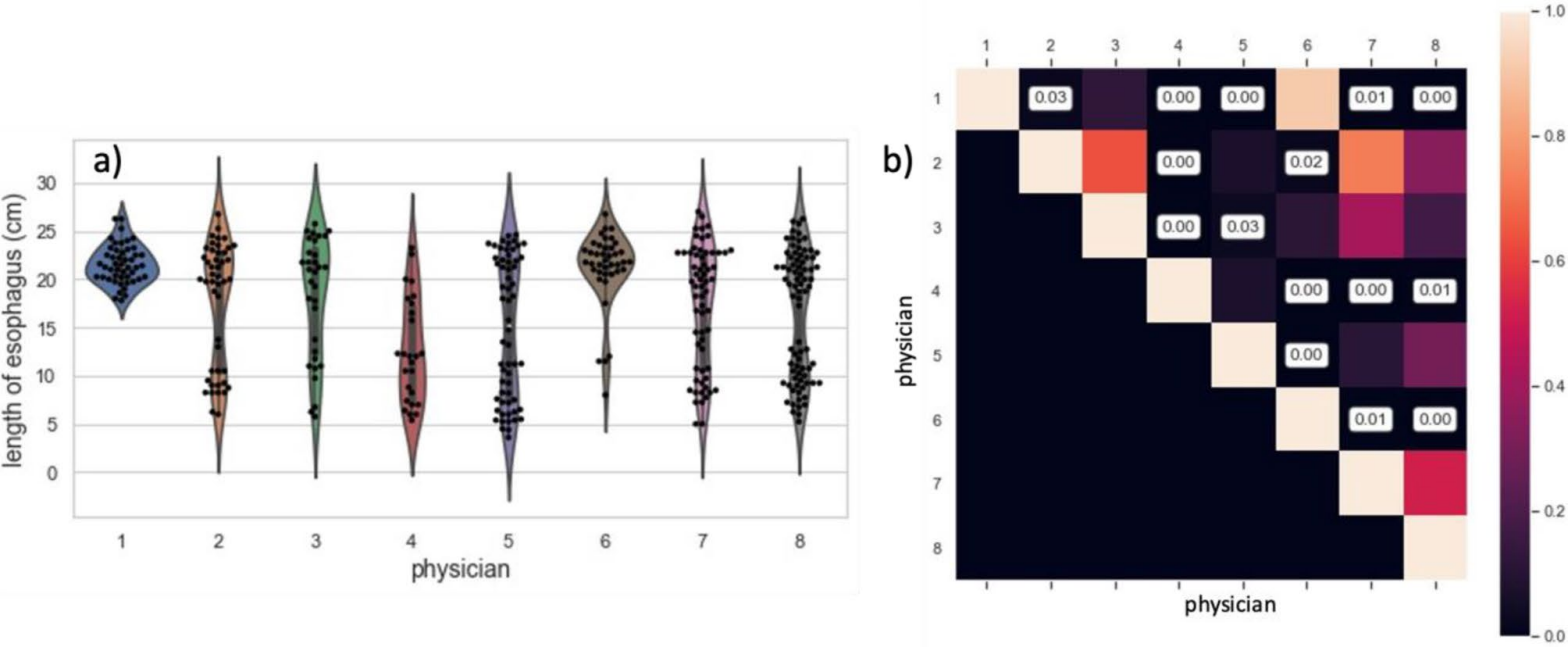
Distribution of length of esophagus segmentations across all physicians (**a**) and *p*-values for pairwise multiple comparison of length of esophagus segmentations (**b**).

The median years of experience for the eight attending radiation oncologists was 9 years (range: 1–17). Figure 2a shows distributions of segmentation length by the eight radiation oncologists. As shown in Supplementary Table 1, the lengths of segmentations by Physician 1 and Physician 6 were in general longer, with a median of 21.3 and 20 cm respectively, and visually consistent with segmenting the entire length of the esophagus. The majority of their lengths was above 18 cm, so we picked this length as a threshold to distinguish full- and partial-length segmentations. The median length of esophageal segmentations by Physician 4 and Physician 5 were smaller at 12.0 and 15.3 cm respectively. In Fig. 2b, the distribution of esophageal length varied significantly between clinicians, demonstrating the intra-observer inconsistency of segmenting the length of the esophagus depending on individual style and clinical context (e.g., for SBRT cases where a point maximum is the critical constraint for the esophagus, the esophagus is often only segmented in the axial slices that overlap with the tumor).

### Metric for clinician's slice-to-slice segmentation inconsistency assessment (Edge Roughness)

We developed a method to quantify a given clinician’s slice-to-slice segmentation inconsistency, using edge roughness—a metric that quantifies the shift of the segmentation among adjacent axial slices. We created 2D projections from sagittal and coronal perspectives by summing up values of the segmentation along the respective axis. We also binarized the 2D projections (i.e., background = 0, segmentation = 1) to capture roughness of jagged edges due to shifted axial slices. We evaluated edge roughness via the concept of local curvature. Local curvature, in mathematics, is used to describe how a curve deviates from a straight line at each local point^17^. Negative and positive curvatures mean more deviation by going into and out of the straight line while zero curvature means a smooth edge. The edge roughness was defined as the sum of the local curvature of the coronal- and sagittal-view projections of the 3-dimensional esophageal segmentation, and divided by the area of the two segmentation surfaces:

$${\text{H}}= -\frac{{((\frac{\partial \sigma }{\partial x})}^{2}+1)*\frac{{\partial }^{2}\sigma }{\partial {y}^{2}}-2*\frac{\partial \sigma }{\partial x}*\frac{\partial \sigma }{\partial y}*\frac{{\partial }^{2}\sigma }{\partial x\partial y}+ {((\frac{\partial \sigma }{\partial y})}^{2}+1)*\frac{{\partial }^{2}\sigma }{\partial {x}^{2}}}{2*{{((\frac{\partial \sigma }{\partial x})}^{2}+ {(\frac{\partial \sigma }{\partial y})}^{2}+1)}^{3/2}}$$

$$\mathrm{Edge Roughness}= \frac{\sum {|H}_{coronal}|+\sum {|H}_{sagittal}|}{{A}_{coronal}+{A}_{sagittal}}$$

where $\sigma (x,y)$ is the representation of a surface patch and H is the curvature map of each point on the corresponding surface. ${H}_{coronal}$ and ${H}_{sagittal}$ represent the local curvature map of segmentation surface on coronal- and sagittal-view projections, respectively. ${A}_{coronal}$ and ${A}_{sagittal}$ represent the surface areas of segmentations on coronal- and sagittal-view projections, respectively. From an anatomy perspective, we would expect a smooth shape of the esophagus. Therefore, a larger edge roughness indicates a greater systematic shift across axial slices.

**Figure 3:**
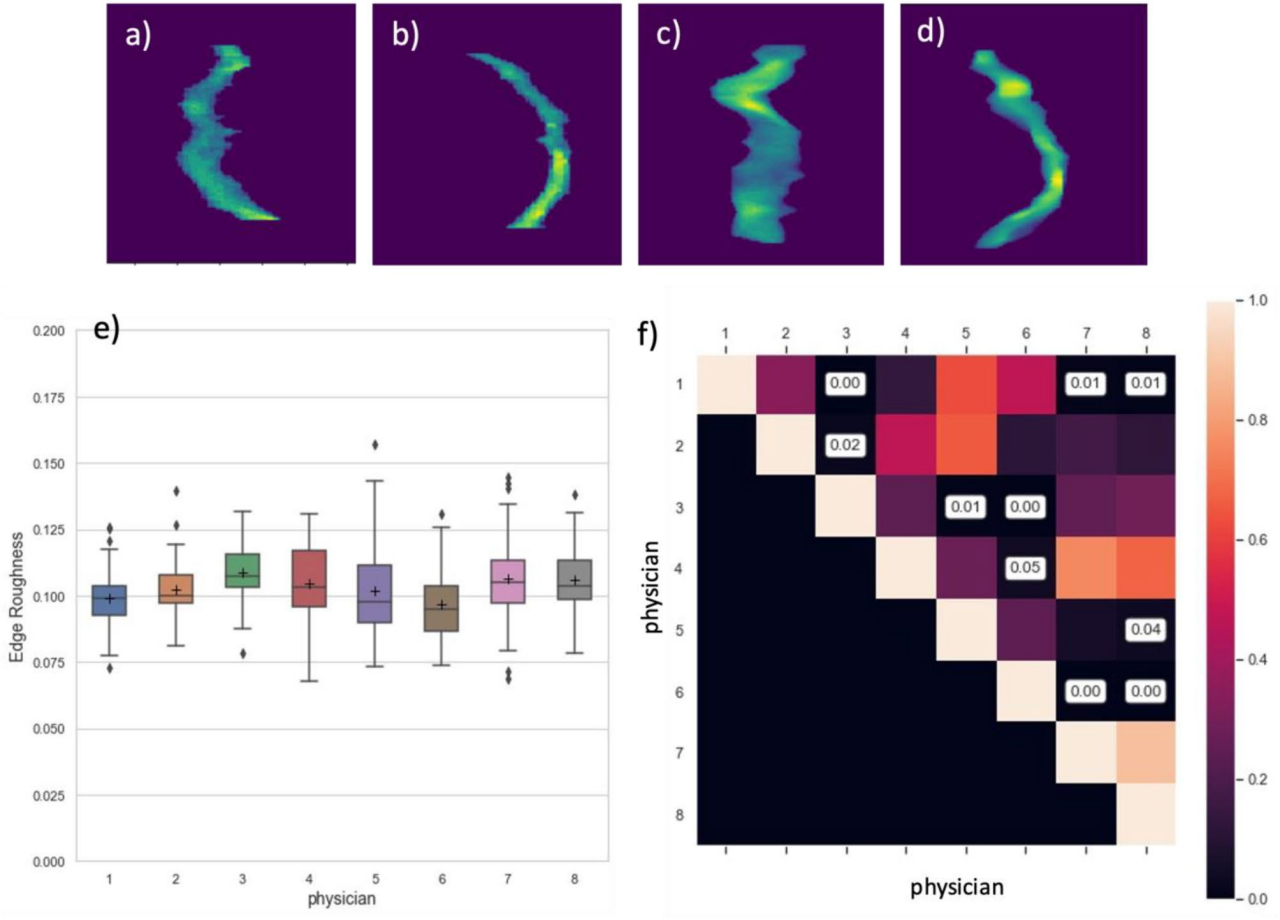
Examples of 2D-projection of the esophagus segmentation: coronal- (**a**, **c**) and sagittal-view (**b**, **d**). (**a**) and (**b**) demonstrate more edge roughness (0.121) and were segmented by physician 7 while (**c**) and (**d**) demonstrate less edge roughness (0.077) and were segmented by physician 1. Distribution of edge roughness across all physicians (**e**) and *p*-values for pairwise multiple comparison of edge roughness (**f**).

For quantitative assessment of clinician inconsistency (Fig. 3e,f), Physician 7’s segmentations had the highest edge roughness (mean ± SD: 0.106 ± 0.016), which was significantly higher than Physician 1’s (mean ± SD: 0.099 ± 0.012, *p* < 0.01) and Physician 6’s (mean ± SD: 0.097 ± 0.013, *p* < 0.01), which can be also visually observed in 2D projections of the esophageal segmentation (Fig. 3a–d).

### Analysis of accuracy of models developed from different training sets (Volumetric Dice Coefficient)

**Figure 4:**
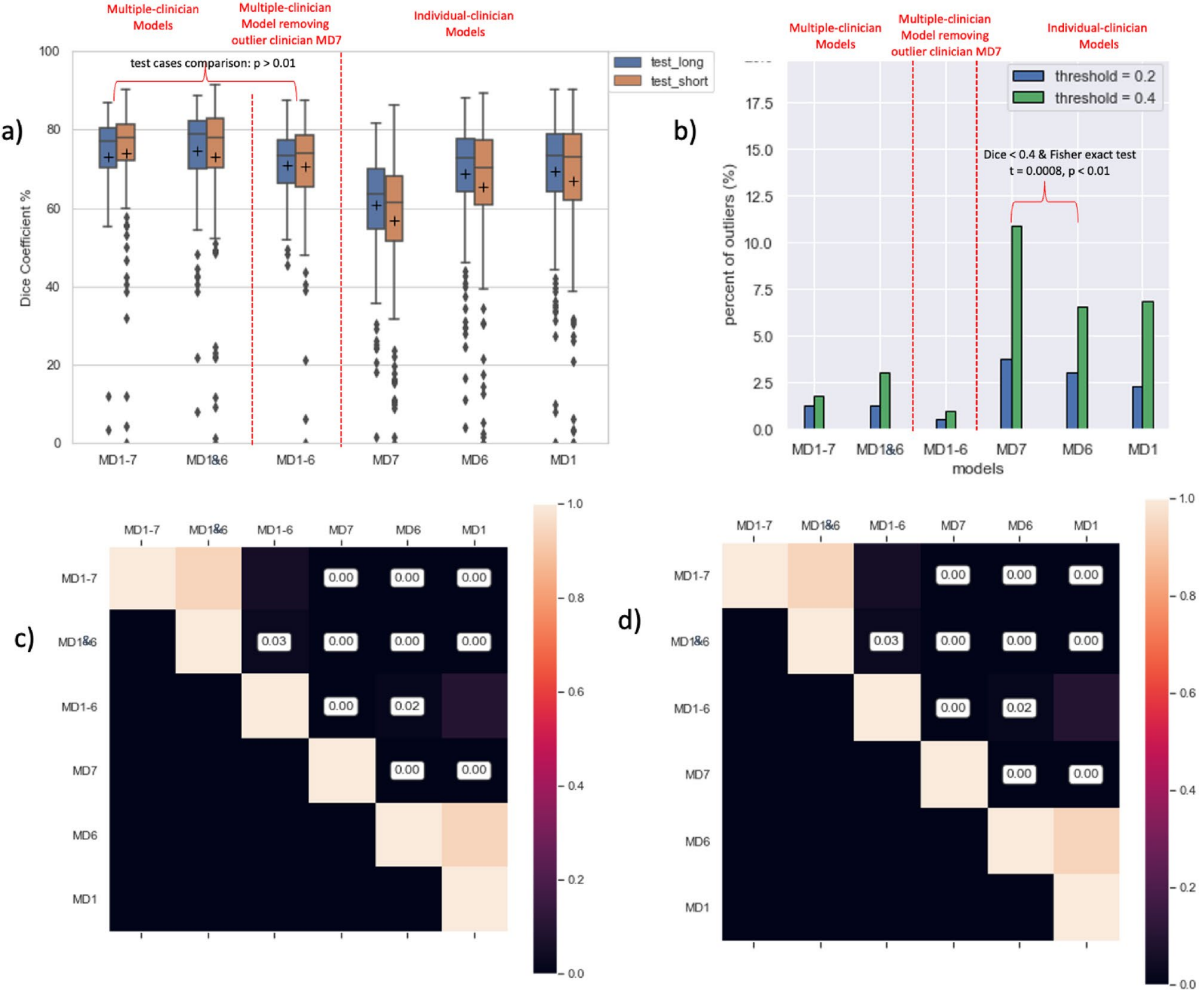
Statistical Summary of Dice coefficient (%) of test data across all models. (**a**) demonstrates the comparison of test cases (both full- and partial-length segmentations) across all six models (see model details in the Method section). (**b**) demonstrates percentage of Dice outliers (over the total case n = 394) across all six models. (**c**) and (**d**) shows the pairwise multiple comparison for full-length and partial-length test data, respectively.

Our primary model, where the training set included all physician’s segmentations (MD1-7), demonstrated a mean Dice of 73.7 ± 14.8% (Fig. 4a & Table S2). There were five outliers in the test set with Dice lower than 20% and seven with Dice lower than 40% (Fig. 4b & Table S4). To improve the model performance, especially by decreasing the number of Dice outliers, we investigated the primary data. We observed more slice-to-slice variations (i.e., jagged edges) in many Physician 7’s segmentations (e.g., Fig. 3a&b) and found a higher mean edge roughness. We then compared performance of models with the outlier physician left out, and individual physician models.

**Figure 5:**
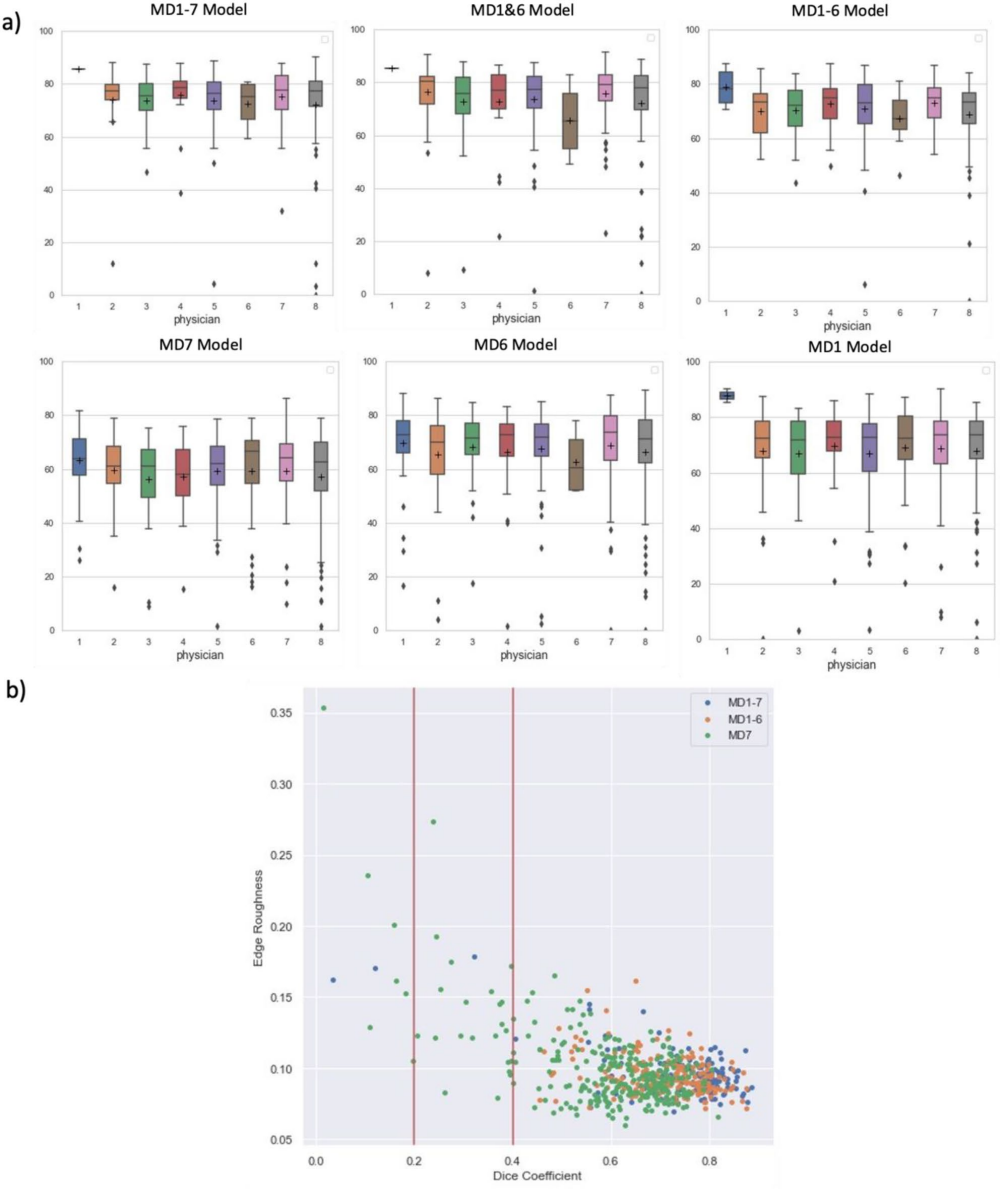
Boxplot of Dice coefficient (%) generated by each model for each physician’s test data (**a**). Edge roughness of prediction vs. Dice coefficient for test data by Model MD1-7, MD1-6 and MD7 (**b**).

To assess the impact of training data quality and inconsistency on model performance, we trained six different models using subsets of the training data: (1) MD1-7 Model: Trained on data from all seven physicians’ segmentations; (2) MD1-6 Model: Excludes training data from outlier Physician 7; (3) MD1&6 Model – training data from the two most consistent physicians; (4) three models trained on three individual physicians (MD7 Model, MD6 Model, and MD1 Model). The mean and standard deviation of Dice coefficients of test data are demonstrated in Fig. 4a (also see Table S2 and S3). For both full-length and partial segmentations in the test set, the model trained on all physicians (MD1-7 Model) and MD1&6 Model outperformed all other models while these two models were not significantly different in terms of Dice coefficients (*p* > 0.01). MD7 Model, which was trained with only Physician 7’s full-length cases, was significantly worse than other models (*p* < 0.01). MD1-6 Model, the model trained without Physician 7’s cases, did not show any significant improvement or decrease in the distribution of Dice coefficient (*p* > 0.01). Distributions of Dice coefficients were also calculated by comparing each model’s segmentation against individual physician’s clinical segmentations (Fig. 5a and Table S3). MD1-7 Model and MD1&6 Model also generated the highest mean Dice coefficients for all physicians across all six models, while MD7 Model, again, had a significantly lower performance (Fig. 4c,d). Figure 5b demonstrated the situation where the predicted segmentation with a lower Dice coefficient tended to have a larger edge roughness. MD7 Model generated a considerable number of Dice outliers (e.g., Dice < 0.4) with corresponding larger edge roughness (e.g., edge roughness > 0.1).

To further support the hypothesis that high edge roughness can result in worse model performance, we trained two additional models using the lowest-quantile edge roughness (MD^ER^-Q1 Model: N_train_ = 62) and highest-quantile edge roughness (MD^ER^-Q4 Model: N_train_ = 62), respectively. The quantiles were calculated based on the distribution of full-length esophagus segmentations (Figure ). These two models were evaluated on the middle 50% of full-length and all partial-length segmentations (N_test_ = 270). The mean and standard deviation of Dice coefficients of the test data, t-statistic, and p-value from t-test are demonstrated in Table 2. MD^ER^-Q1 Model generated a significantly higher mean volumetric Dice coefficient in test cases than the MD^ER^-Q4 Model (mean $\pm$ SD: 67.8 $\pm$ 14.8 vs. 62.8 $\pm$ 15.7, p < 0.0001).

**Table 2:** Comparison of edge roughness and model performance between MD^ER^-Q1 and MD^ER^-Q4.

		MD^ER^-Q1	MD^ER^-Q4
Edge Roughness (N_train_ = 62)	Quantile	0.093	0.108
	mean $\pm$ STD	0.849 ± 0.006	0.118 ± 0.008
	t-statistic	− 24.94	
	*p*-value	1.0e-49	
Volumetric Dice Coefficient (%) (N_test_ = 270)	median	71.0	66.4
	mean $\pm$ STD	67.8 ± 14.8	62.8 ± 15.7
	t-statistic	4.07	
	*p*-value	5.2e-05	

The percent of outliers out of all cases were shown Fig. 4b (also see Table S4 and Figure S1) where four thresholds (i.e., 20%, 40%, 50%, and 70%) of Dice coefficients were chosen. MD1-6 Model, the combined model trained without Physician 7’s cases generated the least outliers when thresholds were 20% and 40%. MD7 Model (N_train_ = 36), even though trained with similar number of cases as MD6 Model (N_train_ = 35), generated significantly more outliers (e.g., Dice ≤ 40%) based on the result of Fisher exact test (t = 0.0008, p < 0.01).

**Figure 6:**
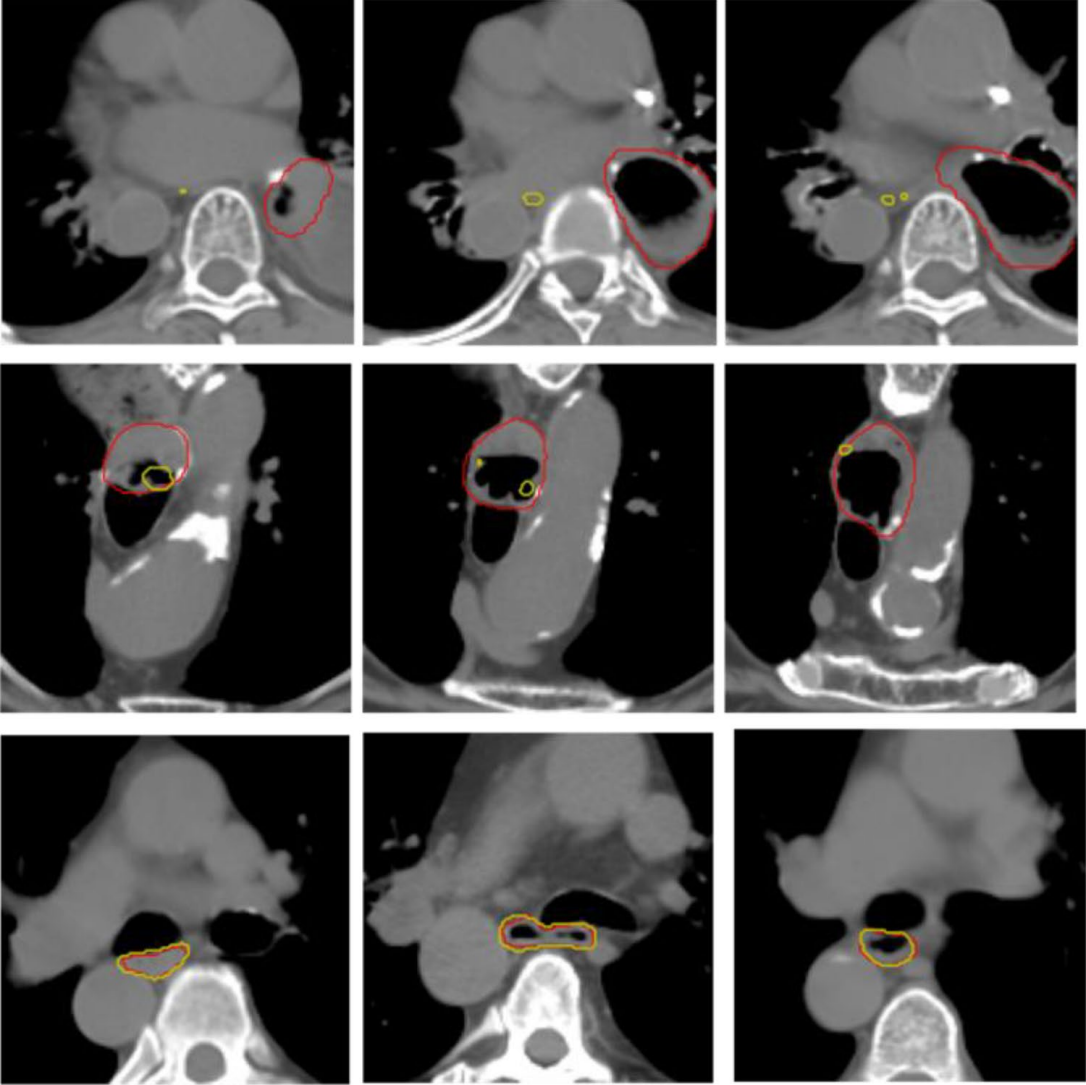
The top two rows are example axial slices for the demonstration of air bubble issue from two patients. The bottom row demonstrates accurate predictions from other patients. (red: ground truth; yellow: model prediction).

Qualitatively, all models had common failure modes, which were predominantly cases with air bubbles in the esophagus (see top two rows in Fig. 6).

## Discussion

Accurate segmentation of tumors and OARs is crucial to RT treatment planning. Deep-learning based segmentation tools can improve the accuracy and efficiency. However, the development of accurate models currently relies on accurate delineation of ground-truth segmentation for model training, which are often generated by expert physicians. Interobserver variation even among experienced physicians can lead to inaccurate or inconsistent ground-truth segmentations. To the best of our knowledge, this is the first study to evaluate the effect of segmentation consistency and style on the deep-learning auto-segmentation of esophagus. Importantly, we developed a novel metric for quantifying segmentation inconsistency and quality by using edge roughness to measure the axial slice-to-slice variation of a segmentation volume, since clinicians typically manually segment CT images on individual axial slices. As hypothesized, our results show a significant decrease in the accuracy and performance of a model trained using segmentations with higher edge roughness. We demonstrated that models that excluded training data with higher edge roughness had a lower frequency of failure (e.g., very low-quality segmentations with Dice < 20%, 40%). These Dice outliers tended to have larger edge roughness. Furthermore, even though the MD1-6 model which excluded the high edge roughness segmentations of MD7, was trained with less data, the overall performance as measured by Dice was still comparable to the benchmark combined model (MD1-7), but with less outliers. While we detected the correlation between edge roughness and model performance in a single physician (MD7), we validated this pattern on the entire dataset. We demonstrated that the model trained with lowest-quantile edge roughness had a significantly better performance than the model trained with highest-quantile edge roughness.

Another innovation of this study is that we propose an evaluation metric for segmentation quality and style in terms of edge roughness given the qualitative observation of the shift of segmentations among axial slices. We used a mathematical concept, curvature, to quantify the roughness of points around segmentation surface edges. This metric can serve as a quantification method of edge roughness for other structures that may involve significant segmentation variation between axial slices. For example, Yang et al.^18^ proposed an approach, Neural Annotation Refinement, to repair distorted segmentations of adrenal gland. Edge roughness can help quantify the distortion of the edge of segmentations which can then be refined by such deep-geometric-learning algorithms.

Observer variation results from various reasons, including each physician’s training, habits, and years of experience. Possible causes of the variation observed in this include habits and training in the use of semi-automated post-processing tools such as interpolation and smoothing functions in the segmentation software versus manual segmentation of every slice, and style. For example, some physicians tend to segment a partial esophagus, depending on the treatment technique (e.g.,SBRT), while others always segment the full-length esophagus in all cases. From a clinical standpoint, there are cases where high accuracy of the esophageal segmentation is more critical, e.g., substantial tumor abutment or proximity, that may have influenced the time spent and quality of the segmentations. Another possible aspect is the use of interpolation functions in software to smooth edges to avoid shift between slices after concatenation into 3D volumes. Some radiation oncologists are trained to use smoothing more often while others use less so that their segmentations tend to shift more among axial slices. While such variation may not have a substantial clinical impact, the development of quantitative metrics such as edge roughness has important implications as a primary quality check to identify and remove outliers prior to algorithm training (or in clinical quality assurance). Thus, the key clinical motivation for this study is that the application of an edge roughness-based filter could benefit subsequent phases of AI auto-segmentation model development by constructing a more consistent training set when using less curated, real-world, clinical data.

We acknowledge several limitations in our study. First, it was difficult to include partial esophagus segmentations in the training set. We have thought about classifying them based on their general locations (e.g., upper, mid, lower esophagus). However, due to the substantial variety of segmented regions, it was challenging to achieve this while avoiding the introduction of more variation. Second, we evaluated Dice coefficient for partial esophagus only based on segmented slices. By excluding partial esophagus in the training set, our model generated full-length predictions, which made it challenging to compare the whole esophagus with its prediction. Additionally, the models all share a common failure mode of producing low-quality segmentations with air bubbles leading to dilated esophagus. We identified five cases with these air bubble artifacts by examining outliers in the model prediction. They were all excluded from the training set due to their shorter length.

In conclusion, our study of training deep-learning segmentation models for esophageal auto-segmentation using data from different individual physician provides evidence of the effect of physician inconsistency, on the performance of auto-segmentation models and identifies a novel metric of inconsistency. The metric, edge roughness, may serve as an evaluation method to identify segmentation inconsistency and data quality check before model development using real-world, clinical data.

## Methods

### Data

We queried the radiation oncology patient database of the Dana-Farber Cancer Institute and Brigham Women’s Hospital for any radiation planning structure sets with an esophagus structure segmented as part of routine clinical care. 574 patients with predominantly lung cancer were selected, each of which had a 3D CT image and an expert-approved esophagus segmentation saved in the respective DICOM RTSTRUCT file. We grouped images of patients based on their corresponding attending physician and selected the top 8 physicians with the most CT scans, resulting in a final study cohort of 394 patients. The study was conducted under a protocol approved by the Dana-Farber/Harvard Cancer Center institutional review board. All methods were performed in accordance with the relevant guidelines and regulations. The study was conducted under a waiver of informed consent approved by institutional review board (protocol Dana-Farber/Harvard Cancer Center 11–286).

### Training and test sets

The lengths of segmented esophagus varied significantly in the real-world data generated by clinicians due to variation in clinical practice depending on radiation therapy technique with full length of the esophagus segmented for conventionally fractionated RT to calculate volumetric exposure (e.g. volume of esophagus receiving 60 Gy or more) versus partial esophageal segmentation at level of the tumor for stereotactic body radiation therapy cases in which point maximum doses to the esophagus are evaluated clinically. Thus, we split the data into full and partial esophagus in terms of length by setting a threshold (L = 18 cm). We picked this length because two physicians (Physician 1&6, MD1&6 Model) who tend to generate more complete segmentations of esophagus per national guidelines (RTOG)^19^. As a result, we ensured enough full-length esophagus cases to train multi-MD models. To have a benchmark model performance, we trained a model with multi-physician data (MD1-7 Model). We also trained a model by excluding Physician 7 as we observed more edge roughness in these cases in our primary quality check (MD1-6 Model). Moreover, we also needed to compare model performances among individual-physician models (MD7 Model, MD6 Model, and MD1 Model).

**Figure 7:**
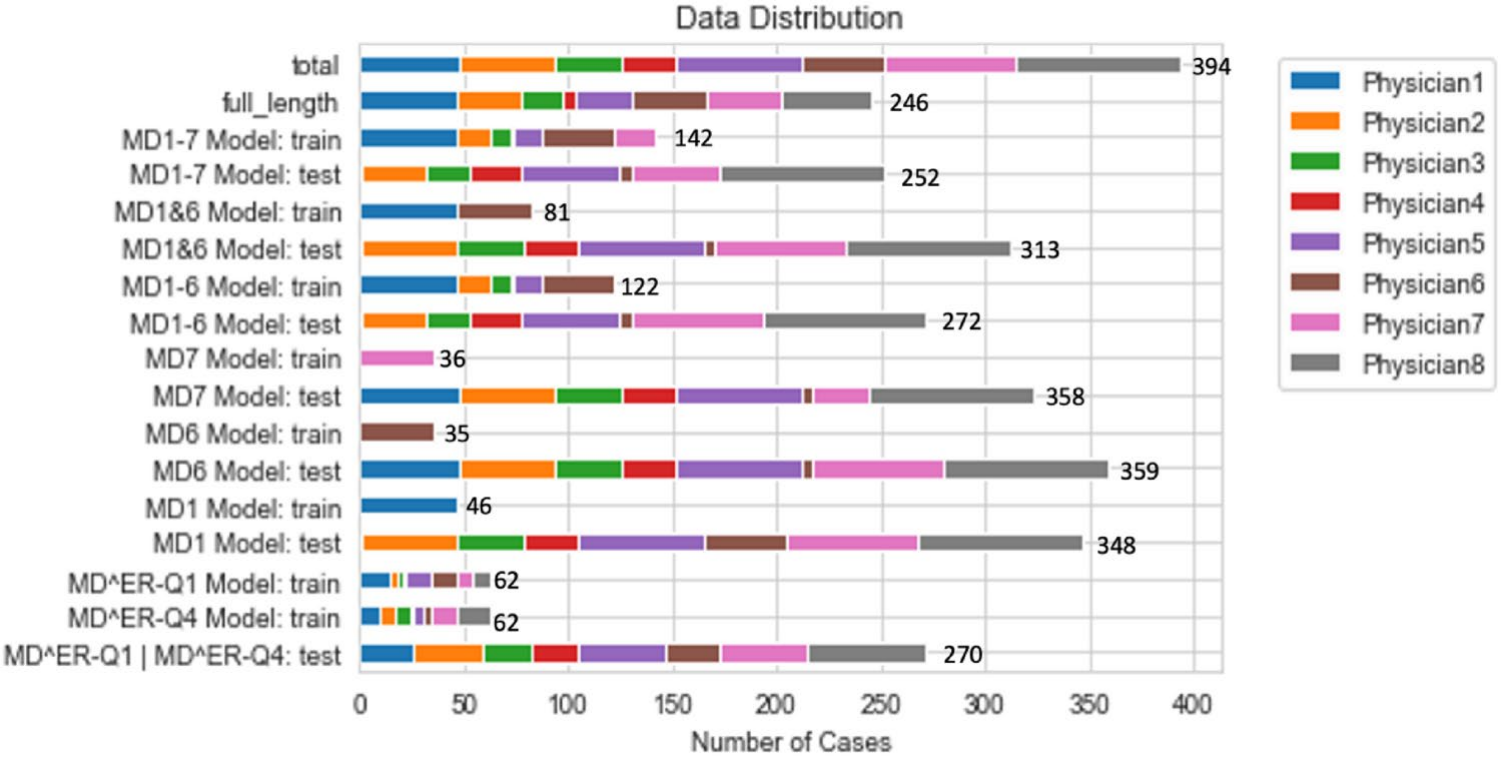
Demonstration of how each physician’s data were distributed among the training and test sets across all eight models.

In the end, according to physicians who made ground-truth segmentations, we constructed six separate models, each of which had the corresponding training data, and the remaining data were used for validation (Fig. 7). Three models were trained and tuned using various combinations of segmentations from multiple physicians (MD1-7 Model, MD1&6 Model, and MD1-6 Model), and the other three of which were trained using images and segmentations from individual physicians (MD7 Model, MD6 Model, and MD1 Model). MD1&6 Model was trained using the combination of full-length segmentations from Physician 1 and Physician 6 (n = 81). MD1-7 Model is composed of training data used in MD1&6 Model as well as randomly sampled segmentations from other five physicians (n = 142). MD1-6 Model was built using data in MD1-7 Model except for the exclusion of Physician 7’s (n = 122) cases. MD7 Model, MD6 Model, and MD1 Model were physician-style-adapted models trained using full-length cases segmented by Physician 7 (n = 36), 6 (n = 35), and 1 (n = 46), respectively. The test set included the segmentations of physician 8 who was intentionally excluded from the training data to have an independent validation dataset, and any data not used in the training set for a given model.

To further confirm the correlation between edge roughness and model performance, we also trained two models based on the quantile of edge roughness distribution, one’s training set containing only the lowest-quantile edge roughness and the other containing the highest-quantile edge roughness (MD^ER^-Q1 Model: n = 62 and MD^ER^-Q4 Model: n = 62). The rest of data were used as the test set (n = 270) to evaluate the two models.

### Deep-learning models for automatic segmentation

We developed a deep-learning semantic segmentation system to automatically localize and segment the esophagus from a CT scan and using expert segmentations generated during clinical care as ground truth training data. The proposed system consists of two steps (i.e., localization and segmentation), each of which used a 3-dimensional U-Net architecture with residual blocks as backbone (Fig. 1). The encoder-decoder structure follows the original implementation^20^. The residual block consists of two residual layers that involve batch normalization, 3D convolution, and ReLU activation (Fig. 1). In the step of localization, we reduced the size of the original 3D CT volume and segmentation and used them to train a localizer to identify the rough location of the esophagus^21^. We then mapped the compressed volume back to its original size and acquired the predicted coordinates of the mapped esophagus.

After resampling the original volume and segmentation with a voxel spacing of 1 mm, we applied the predicted coordinates onto the resampled volume to localize the esophagus and expanded the bounding box to the size of 128 × 128 × 128 pixels. To increase the contrast between the target structure and neighbors and speed up training, we applied a CT window (− 340 < HU < 460), followed by intensity normalization, to the selected sub-volume that contains the esophagus and the surrounding tissue.

Data augmentation methods were adopted to enrich the complexity of training data, including random flipping, rotation, translation, and scaling. Separate models with different combinations of images and segmentations were trained and tuned using the residual U-Net. Predicted segmentations were generated and processed to remove small fragments. Final predictions were evaluated by volumetric Dice Coefficient. All models were trained on a NVDIA GPU with 32 GB of memory, using TensorFlow 2.2. The batch size was set to 4, the learning rate was 0.01 and the validation split was 0.2.

### Accuracy assessment

The accuracy of AI segmentation of the esophagus was evaluated by using the volumetric Dice coefficient with physician’s segmentations as ground truth. The volumetric Dice coefficient^22^ is defined as:

$$\mathrm{Dice Coefficient }=\frac{2\left({V}_{p}\cap {V}_{GT}\right)}{{V}_{p}+{V}_{GT}}$$

where ${V}_{p}$ represents the predicted volume by AI models and ${V}_{GT}$ represents the ground truth segmentation volume created by physicians. Since we trained models using full-length segmentations, for partial-length segmentations, we only evaluated the volumetric Dice only within regions where ground truth labels existed.

### Statistical analysis

*P*-values for the comparison of edge roughness, length of segmentations, and Dice coefficients were generated from the Dunn’s test, a non-parametric pairwise multiple comparison test following the rejection of an ANOVA null hypothesis^23,24^. Proportions in Dice outliers were compared using Fisher exact test^25^. *P*-values were two-sided and considered significant below 0.01.

### Supplementary Information

Supplementary Information.

## Data Availability

The study was conducted under a protocol approved by the Dana-Farber/Harvard Cancer Center institutional review board. Due to privacy agreements with our institutions, the datasets generated and/or analyzed during the current study are not publicly available but are available from the corresponding author on reasonable request.
