# Supplementary material for: Edge roughness quantifies impact of physician variation on training and performance of deep learning auto-segmentation models for the esophagus
Source: Sci Rep. 2024 Jan 30;14:2536. doi: 10.1038/s41598-023-50382-z (PMC10827712; doi:10.1038/s41598-023-50382-z)
Supplement: Supplementary file 1 — Supplementary Information. [file 41598_2023_50382_MOESM1_ESM.docx]

**Supplementary Material**

**Table S1. Summary of length of segmentations and number of full-length cases in each physician’s group**

| Radiation Oncologist | Length of Segmentation (centimeter)  Median (25%, 75%) | Number of Cases (full-length)  (total = 394, full-length = 245) |
| --- | --- | --- |
| (1) | 21.3 (20.2, 22.8) | 48 (46) |
| (2) | 20 (10.5, 22.8) | 46 (30) |
| (3) | 20.9 (12.3, 23.1) | 32 (20) |
| (4) | 12.0 (7.6, 17.3) | 26 (6) |
| (5) | 15.3 (7.4, 22.1) | 60 (28) |
| (6) | 21.8 (20.8, 23.3) | 40 (35) |
| (7) | 19.3 (10.8, 22.8) | 63 (36) |
| (8) | 19.3 (9.9, 22.1) | 79 (44) |

**Table S2. Dice coefficient of overall training and test sets**

| Mean Dice Coefficient ± SD (%) | | | | | | |
| --- | --- | --- | --- | --- | --- | --- |
|  | **MD1-7 Model** | **MD1&6**  **Model** | **MD1-6**  **Model** | **MD7**  **Model** | **MD6**  **Model** | **MD1**  **Model** |
| Training | 82.1 ± 8.5  (n = 142) | **87.8 ± 7.7**  **(n = 81)** | 80.1 ± 6.8  (n = 122) | *69.0 ± 8.6*  *(n = 36)* | 81.0 ± 13.0  (n = 35) | 84.4 ± 9.6  (n = 46) |
| Test: full-length | 73.0 ± 13.6  (n = 74) | **74.7 ± 12.1**  **(n = 165)** | 71.0 ± 8.7  (n = 124) | *60.7 ± 13.1*  *(n = 210)* | 68.9 ± 13.9  (n = 211) | 69.3 ± 15.3  (n = 200) |
| Test: partial | **74.0 ± 15.4**  **(n = 148)** | 72.4 ± 18.2  (n = 148) | 70.8 ± 14.5  (n = 148) | *56.1 ± 18.7*  *(n = 148)* | 64.6 ± 20.0  (n = 148) | 66.3 ± 19.5  (n = 148) |
| Test: both | **73.7 ± 14.8**  **(n = 252)** | 73.9 ± 15.3  (n = 313) | 70.9 ± 12.2  (n = 272) | *58.8 ± 15.8*  *(n = 358)* | 67.1 ± 16.8  (n = 359) | 68.0 ± 17.3  (n = 348) |

*bold: the highest value in the current row/category; italic: the lowest value in the current row/category

**Table S3. Dice coefficient generated by each model for each physician’s test data**

| Mean Dice Coefficient ± SD (%) | | | | | | |
| --- | --- | --- | --- | --- | --- | --- |
|  | **MD1-7**  **Model** | **MD1&6**  **Model** | **MD1-6 Model** | **MD7**  **Model** | **MD6**  **Model** | **MD1**  **Model** |
| Physician 1 | 84.3 ± 4.9  (n = 2) | **85.2 ± 7.8**  **(n = 2)** | 78.8 ± 5.9  (n = 2) | *63.1 ± 11.6*  *(n = 48)* | 69.7 ± 11.7  (n = 48) | 84.6 ± 3.4  (n = 2) |
| Physician 2 | **77.0 ± 12.3**  **(n = 31)** | 76.3 ± 13.3  (n = 46) | 70.0 ± 9.7  (n = 31) | *59.6 ± 12.5*  *(n = 46)* | 65.5 ± 10.4  (n = 46) | 67.8 ± 18.8  (n = 46) |
| Physician 3 | **76.4 ± 9.3**  **(n = 21)** | 72.9 ± 15.0  (n = 32) | 70.3 ± 9.1  (n = 21) | *56.3 ± 15.8*  *(n = 32)* | 68.1 ± 10.4  (n = 32) | 66.9 ± 16.5  (n = 32) |
| Physician 4 | **75.5 ± 11.3**  **(n = 25)** | 72.9 ± 15.3  (n = 26) | 72.8 ± 8.9  (n = 25) | *57.1 ± 14.0*  *(n = 26)* | 66.5 ± 11.8  (n = 26) | 69.7 ± 14.5  (n = 26) |
| Physician 5 | **75.2 ± 14.0**  **(n = 47)** | 73.8 ± 14.0  (n = 60) | 70.9 ± 12.9  (n = 47) | *59.1 ± 13.9*  *(n = 60)* | 67.7 ± 10.7  (n = 60) | 66.8 ± 16.8  (n = 60) |
| Physician 6 | **84.6 ± 7.9**  **(n = 5)** | 65.6 ± 12.9  (n = 5) | 67.1 ± 8.8  (n = 5) | *59.4 ± 17.5*  *(n = 40)* | 62.7 ± 12.7  (n = 5) | 69.2 ± 15.5  (n = 40) |
| Physician 7 | **77.4 ± 10.4**  **(n = 43)** | 75.7 ± 11.7  (n = 63) | 71.2 ± 9.2  (n = 63) | *59.3 ± 14.2*  *(n = 27)* | 68.7 ± 13.6  (n = 63) | 68.9 ± 16.4  (n = 63) |
| Physician 8 | 72.0 ± 16.5  (n = 79) | **72.3 ± 17.6**  **(n = 79)** | 68.9 ± 13.4  (n = 79) | *57.2 ± 18.5*  *(n = 79)* | 66.4 ± 12.8  (n = 79) | 68.0 ± 18.2  (n = 79) |

*bold: the highest value in the current row/category; italic: the lowest value in the current row/category

**Table S4. Outliers of Dice Coefficient for each model**

| Percent of Dice Coefficient (DC) Outliers (%)  (Number of Cases, Total = 394) | | | | | | |
| --- | --- | --- | --- | --- | --- | --- |
|  | **MD1-7 Model** | **MD1&6**  **Model** | **MD1-6**  **Model** | **MD7**  **Model** | **MD6**  **Model** | **MD1**  **Model** |
| DC <= 20% | 1.3 (5) | 1.3 (5) | **0.5 (2)** | 3.8 (15) | 3.0 (12) | 2.3 (9) |
| DC <= 40% | 1.8 (7) | 3.0 (12) | **1.0 (4)** | 10.9 (43) | 6.6 (26) | 6.8 (27) |
| DC <= 50% | **3.5 (14)** | 5.6 (22) | **3.5 (14)** | 18.8 (74) | 9.9 (39) | 10.1 (40) |
| DC <= 70% | **15.7 (62)** | 20.8 (82) | 27.4 (108) | 73.1 (288) | 41.1 (162) | 33.9 (134) |

*bold: the lowest percentage (i.e., best model) in the current row/category

**Table S5. Data Distribution for MD^ER^-Q1 Model, MD^ER^-Q4 Model, and the Common Test Set**

| **Physician** | **Count (n_train_ = 62)**  **MD^ER^-Q1** | **Count (n_train_ = 62)**  **MD^ER^-Q4** | **Count (n_test_ = 270) test** |
| --- | --- | --- | --- |
| **Physician 1** | 14 | 9 | 25 |
| **Physician 2** | 4 | 8 | 34 |
| **Physician 3** | 2 | 7 | 23 |
| **Physician 4** | 2 | 1 | 23 |
| **Physician 5** | 12 | 6 | 42 |
| **Physician 6** | 13 | 3 | 24 |
| **Physician 7** | 7 | 13 | 43 |
| **Physician 8** | 8 | 15 | 56 |

*
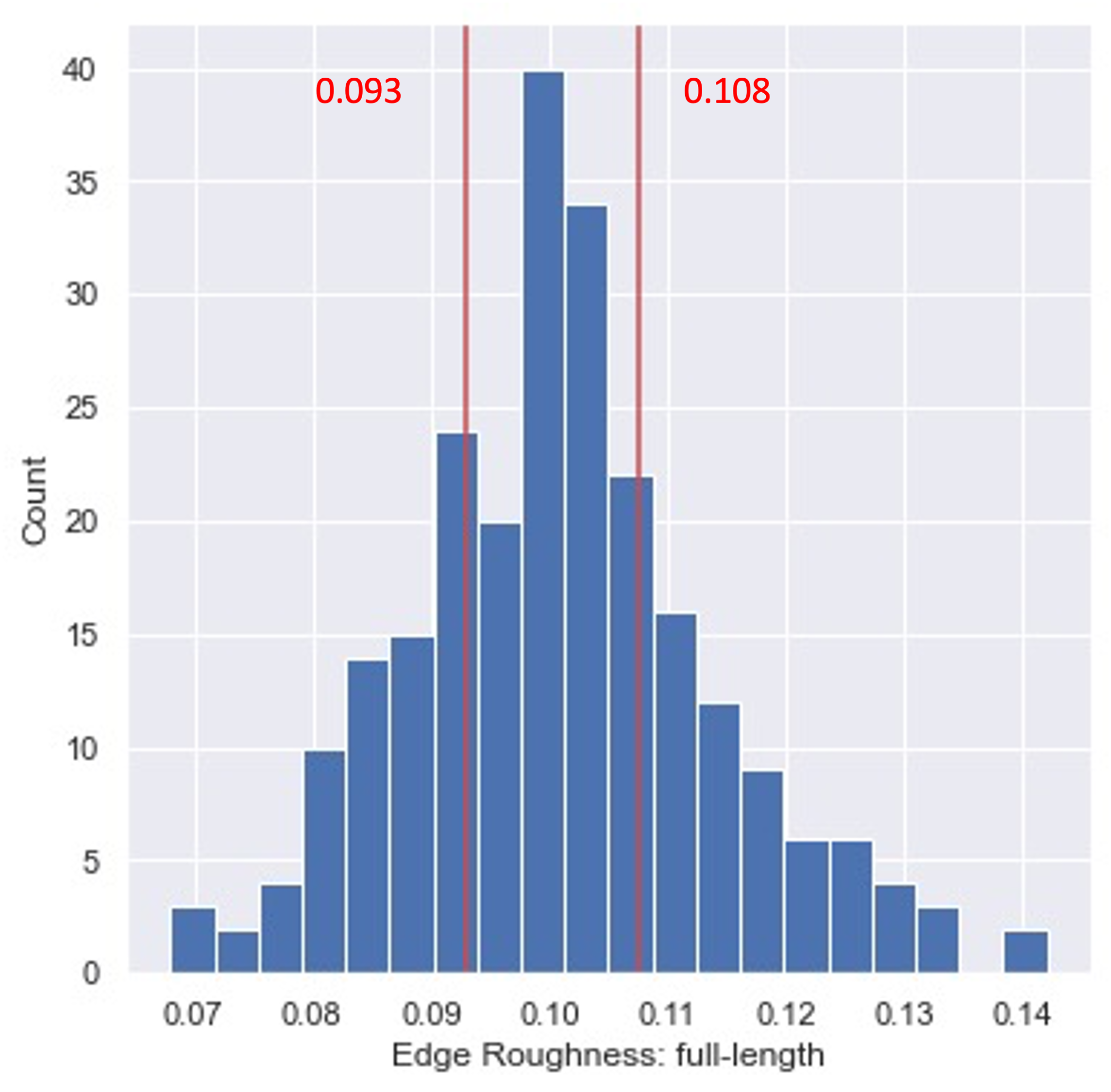
*

*Figure S1. Distribution of Edge Roughness of Full-length Esophagus Segmentation*

*
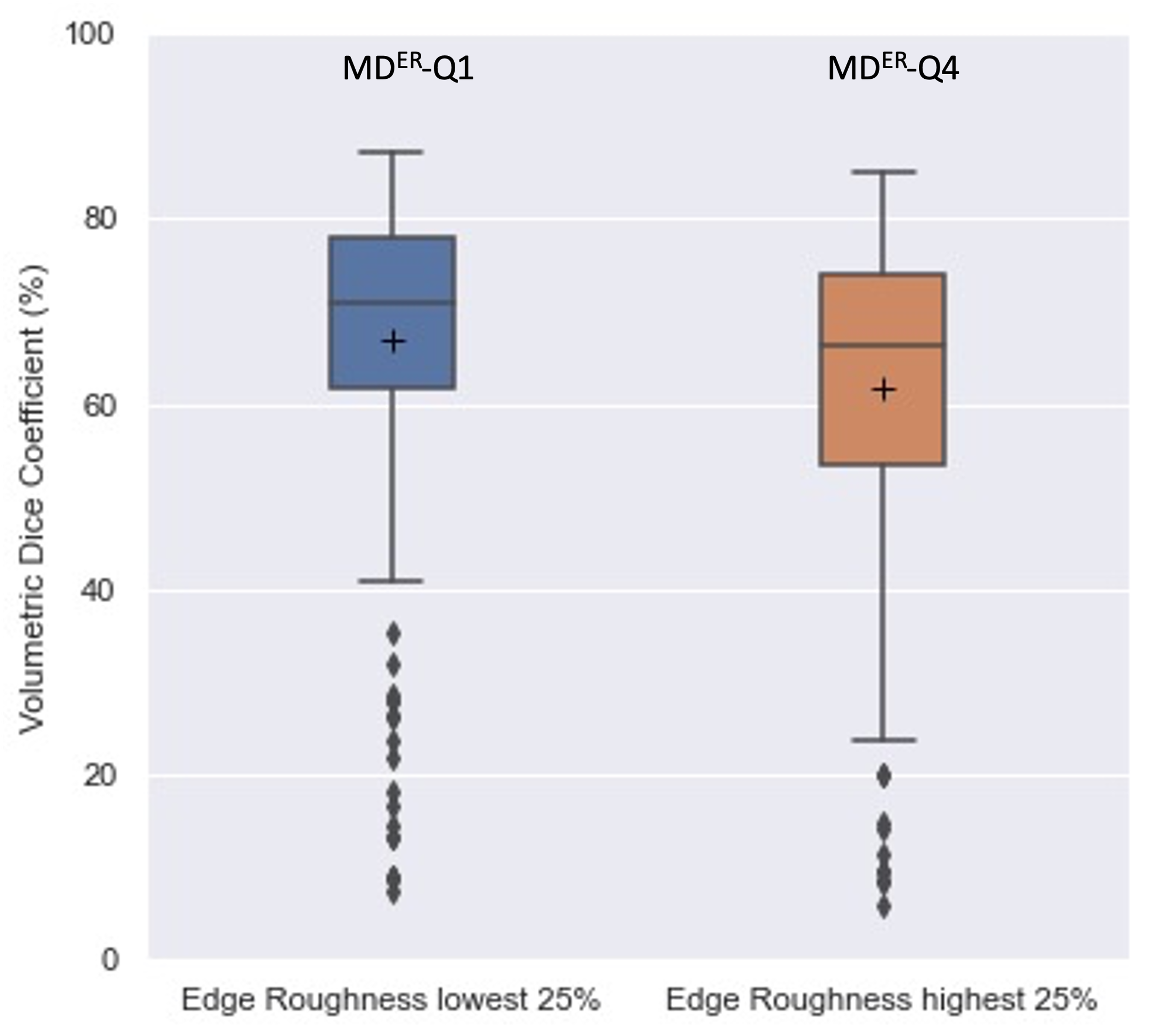
*

*Figure S2. Comparison of Model Performance between MD^ER^-Q1 and MD^ER^-Q4*

**
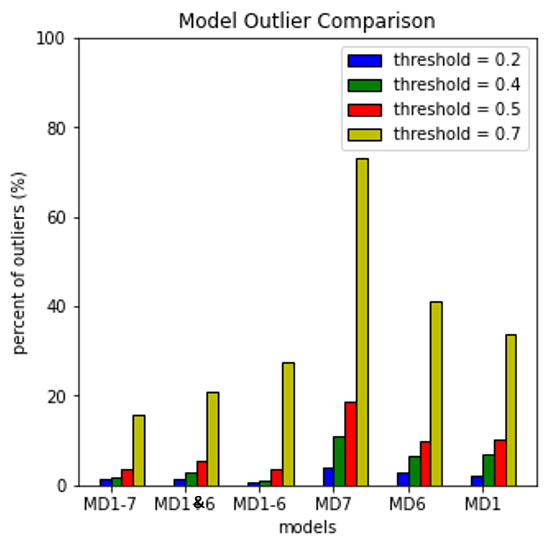
**

*Figure S3. Bar plot of Outliers of Dice Coefficient for each model (see Table4)*
